# Editorial: Microsurgical anatomy of the central nervous system and skull base volume II

**DOI:** 10.3389/fsurg.2025.1593925

**Published:** 2025-04-11

**Authors:** Alexander I. Evins, Gustavo R. Isolan

**Affiliations:** ^1^ Department of Neurological Surgery, Weill Cornell Medicine/NewYork-Presbyterian Hospital, New York, NY, United States; ^2^ Centro Avançado de Neurologia e Neurocirurgia (CEANNE), Porto Alegre, Brazil

**Keywords:** surgical anatomy, neurosurgery, central nervous system, microneurosurgery, neurosurgical approaches

**Editorial on the Research Topic**
Microsurgical anatomy of the central nervous system and skull base volume II

In the realm of human anatomy, the central nervous system stands out as an intricate labyrinth of deep pathways, giving rise to networks of neurovasculature—nowhere more notably than along the base of the skull where critical structures join together to form intricate three-dimensional neurovascular complexes. This detailed anatomical architecture reflects a complexity that inspires exploration, beckoning us as surgeons to venture beyond mere surface exposure in order to access lesions embedded in the deepest recesses of the cranium and, in doing so, illuminate the structures that define our patients' abilities to see, hear, smell, and sense the world. As surgeons, we are guided by an insatiable curiosity and a steadfast commitment to precision. We delve into the fine details of anatomy, recognizing that these structures—their topography and spatial relationships—provide both a challenge and an opportunity to refine our surgical approaches and deepen our curative potential.

Accordingly, we are pleased to present the second volume of this special Research Topic on *Microsurgical anatomy of the central nervous system and skull base*. The work included in this topic expands our collective insight, further highlighting the importance of anatomical dissection in neurosurgery and demonstrating the value of this research for improving patient care and outcomes (1–5).

The study by Oğlin et al. examines the topographic variations of the lateral parietal cortex and their relationship to the underlying white matter tracts utilizing fiber dissection of 28 cadaveric hemispheres along with MRI tractography data. The study reveals critical relationships between parietal sulci and major white matter pathways, finding that the postcentral and intraparietal sulci were continuous in most specimens (71% right, 64% left) and that more lateral positioning of their meeting point increased the risk of damaging the arcuate fasciculus and superior longitudinal fasciculus II during neurosurgical approaches to deep parietal and atrial lesions.

Ćetković et al. explores the existence of a relationship between cerebral artery fenestrations and aneurysms. The authors combined microsurgical dissection of 50 specimens with a retrospective review of computed tomography angiograms from 1,230 patients and found fenestrations in 24% of anatomical specimens and 2.11% of clinical cases, while aneurysms were observed in 12% and 2%, respectively. Ultimately, the association between fenestrations and co-located aneurysms proved exceedingly rare (2% in anatomical specimens and 0.08% in clinical cases arising from the fenestration), suggesting that although fenestrations are developmental remnants, they seldom coincide with aneurysm formation.

The study by Sousa et al. proposes a standardized approach for free-hand endoscopic third ventriculostomy by defining a novel optimal endoscope trajectory for accessing the floor of the third ventricle in the setting of hydrocephalus. Analyzing 187 cranioencephalic MRIs, including 30 with hydrocephalus, the authors measured distances to key cranial sutures and anatomical landmarks as well as pathway angles and depths. They report significant differences in parameters such as the depth to the tuber cinereum, coronal plane angulation, and distance to the sagittal suture between hydrocephalic and control groups. The findings suggest that an entry point located near the cranial sutures and away from the precentral and superior frontal sulci could enhance surgical precision and outcomes, offering a useful technique in emergency settings or when specialized navigation tools are unavailable.

Liang et al. investigate the impact of excising the medial wall of the cavernous sinus during endoscopic endonasal transsphenoidal resection of functioning pituitary adenomas. In a retrospective analysis of 41 patients, the study found that medial wall excision resulted in significantly higher biochemical remission rates (75% vs. 38%), while complication and recurrence rates remained consistent between groups. Histopathological confirmation of tumor invasion into the medial wall was found in 71% of cases. These findings suggest that endoscopic excision of the medial wall of the cavernous sinus could be a safe and effective adjunct for transsphenoidal management of functioning pituitary adenomas with suspected invasion into the cavernous sinus.

The study by Ye et al. investigates the surgical anatomy and morphology of the bridging veins coming off of the superior sagittal sinus encountered in interhemispheric approaches. Using a combination of cadaveric dissection and magnetic resonance venography from 86 patients, the authors classify bridging vein configurations into three types. Histological staining further corroborated these categories, revealing variations in collagen fiber arrangement and tissue composition. This classification provides surgeons with a practical framework to predict bridging vein configuration and tailor dural incisions accordingly in order to maximize intraoperative exposure while minimizing the risk of venous injury during interhemispheric approaches.

Shiferaw et al. examine predictors of mortality following skull base tumor resections in Ethiopia through a retrospective cohort study of 266 patients and reveal a high 3-month mortality rate of 21.1%. The authors identify four significant independent risk factors for mortality, including intraoperative iatrogenic vascular insult, intraventricular hemorrhage, hospital-associated infection, and extubation time exceeding 24 h. These findings underscore the critical need for meticulous preoperative training in skull base techniques to improve surgical outcomes in resource-limited neurosurgical settings. These findings highlight the urgent need for improved neurosurgical infrastructure, training, and perioperative care in low-income settings to address the significant disparity in mortality rates compared to higher-income countries where mortality for similar procedures is approximately 0%–5%.

Finally, the study by Isolan et al. investigates the predictive value of their proposed “Porto Alegre Line”—a novel MRI landmark—for identifying lenticulostriate artery encasement by insular gliomas. In a retrospective review of 52 insular glioma patients, the authors demonstrated that lenticulostriate artery involvement was the most critical factor limiting resection of medial tumor, with a significant impact on overall survival. When validated against intraoperative findings, the Porto Alegre Line showed excellent predictive value with sensitivity and specificity, confirming it as a reliable indirect imaging indicator for lenticulostriate artery involvement when tumors extended medially beyond the line. These findings suggest that incorporating the Porto Alegre Line into preoperative planning could enhance surgical planning and decision-making by identifying patients at high risk for lenticulostriate artery encasement, potentially improving outcomes in insular glioma surgery.

We hope that the readership will find these articles and this research topic to be a useful source for emerging anatomically-based research in the clinical neurosciences.
